# Multi-Material Additive Manufacturing for Advanced High-Tech Components

**DOI:** 10.3390/ma15186433

**Published:** 2022-09-16

**Authors:** Flávio Bartolomeu, F. S. Silva

**Affiliations:** 1 Center for MicroElectroMechanical Systems (CMEMS-UMinho), Campus de Azurém, University of Minho, 4800-058 Guimarães, Portugal; 2 LABBELS–Associate Laboratory, Braga/Guimarães, Portugal

***Multi-Material Additive Manufacturing for Advanced High-Tech Components*** is a new open Special Issue of *Materials*, which aims to publish original and review papers regarding new scientific and applied research and make great contributions to finding, exploring and understanding novel multi-material components via additive manufacturing.

The development of multi-material components able to address a high level of multi-functionality based on distinct materials intrinsic properties might be a method of achieving unparalleled solutions for a wide range of applications. By exploiting the potential of additive manufacturing potential, new 3D multi-material solutions can be created for medical implants, aerospace components, molds, jewelry, collection coins and many other applications. Using advanced manufacturing technologies, such as laser powder bed fusion (adapted for manufacturing with different powders in 3D), different materials can be combined in a single component such as steel and copper, steel and titanium, Inconel and copper, Inconel and aluminum, titanium and cobalt, titanium and tantalum, titanium and zirconia, steel and silver, silver and gold, silver and copper, etc. 

The combination of different materials in a single component can bring to life new possibilities, never studied, with tailored and combined functions (mechanical, physical or chemical properties). This technology and disruptive design philosophy are being investigated at CMEMS-UMinho, in Guimaraes–Portugal. A novel 3D multi-material design concept targeting plastic injection moulds was proposed by A. Cunha et al. [1]. This multi-material component combines the mechanical resistance of the 420 stainless steel alloy and the high thermal conductivity of copper, in a single component, fabricated in just one event by means of home-made 3D Multi-Material Laser Powder Bed Fusion equipment. Also, A. Marques et al. [2] fabricated novel Inconel 718–copper parts by 3D multi-material laser powder bed fusion targeting rocket engines. Topics of interest for this Special Issue, entitled Multi-Material Additive Manufacturing for Advanced High-Tech Components, includes, but is not limited to, the following (reviews and experimental and numerical studies are welcome): advanced additive manufacturing strategies; laser powder bed fusion; metal-based multi-material design; nature-inspired architectures and solutions via AM; multi-functional components; smart materials; topological optimization and high-efficiency solutions.
